# Commentary on “Re-exploration of the optimal threshold for restrictive transfusion after hip fracture: a multicenter prospective cohort”

**DOI:** 10.1097/JS9.0000000000003157

**Published:** 2025-08-06

**Authors:** Jicheng Li, Jian Tan, Xing Du

**Affiliations:** a Department of Orthopaedics, Dazhou Dachuan District People’s Hospital (Dazhou Third People’s Hospital), Dazhou, China; b Department of Orthopaedic Surgery, The First Affiliated Hospital of Chongqing Medical University, Chongqing, China; c Chongqing Municipal Health Commission Key Laboratory of Musculoskeletal Regeneration and Translational Medicine, Chongqing, China

*Dear editor,*

Jiang *et al* conducted a prospective multicenter cohort study for assessing whether temporarily lowering the red blood cell transfusion threshold from hemoglobin (Hb) < 8 g/dL (standard care) to <7 g/dL (alternative care during blood shortages) is safe in patients ≥65 years undergoing hip fracture surgery^[1]^. They found the overall one-year mortality was similar between groups, however, in patients with baseline cardiovascular disease (CVD), mortality was significantly higher with the <7 g/dL threshold. The authors concluded that using a <7 g/dL threshold appears safe for patients without CVD during blood shortages, but the standard <8 g/dL threshold should be maintained for those with CVD. Despite the strengths inherent in its prospective design, multicenter recruitment, large sample size, and sophisticated statistical modeling, several significant limitations constrain the interpretation and generalizability of the findings. In line with the TITAN 2025 guideline on AI transparency^[2]^, not any AI tools was used in this research or manuscript development.

The principal limitation arises from the observational cohort design. While the study rigorously adjusted for numerous measured confounders, residual confounding due to unmeasured or imperfectly measured factors remains highly probable and impactful^[3,4]^. Crucially, comprehensive markers of frailty (e.g., nutritional status, sarcopenia severity, cognitive reserve)^[5,6]^, and variations in postoperative rehabilitation quality^[7]^, were not systematically assessed. This omission raises the possibility that patients maintaining lower Hb levels inherently possessed better physiological reserve, while clinicians may have preferentially transfused frailer patients (confounding by indication)^[3,8]^ -a bias inherently resistant to full statistical correction in observational studies^[4]^.

A significant methodological concern relates to hemoglobin measurement. Hb was assessed periodically, failing to capture critical transient nadirs and variability between measurements^[9]^. Consequently, patients experiencing brief, severe Hb drops (e.g., to 6.5 g/dL) recovering by the next scheduled test could be misclassified. Furthermore, the exposure variable (maintained Hb level) results from the interplay of clinician trigger thresholds, bleeding, and physiology, not a standardized intervention^[9,10]^. The absence of reported specific trigger Hb values makes it difficult to equate the observed maintained level outcomes with testing a specific intervention threshold as in an randomized controlled trial (RCT)^[10]^.

Finally, the study neglects the symptom burden of anemia. Without assessing Patient-Reported Outcomes (PROs) related to low Hb levels (e.g., profound fatigue impacting rehabilitation tolerance, breathlessness limiting mobility, anxiety)^[6,7,11]^, the analysis omits a crucial dimension of patient well-being. A strategy reducing mortality but causing frequent, debilitating symptoms may be unacceptable despite survival benefits – a risk-benefit trade-off left unexplored.

In conclusion, though this study provides valuable observational evidence for the optimal transfusion thresholds in hip fracture patients, inherent methodological limitations preclude definitive practice changes. Future research should incorporate orthopedics-specific functional assessments, comprehensive PROs (symptoms, quality of life), and dedicated analysis of high-risk subgroups (e.g., CVD patients) to conclusively establish safety, efficacy, and optimal transfusion thresholds across this heterogeneous population, ultimately transforming evidence into clinically actionable guidance.

## Data Availability

No new data were generated or analyzed.

## AI assistance

Not any AI tools was used in the research or manuscript development.
